# *Mycoplasma suis* infection results endothelial cell damage and activation: new insight into the cell tropism and pathogenicity of hemotrophic mycoplasma

**DOI:** 10.1186/1297-9716-44-6

**Published:** 2013-02-11

**Authors:** Albina Sokoli, Katrin Groebel, Katharina Hoelzle, Werner M Amselgruber, José M Mateos, Mårten KJ Schneider, Urs Ziegler, Kathrin M Felder, Ludwig E Hoelzle

**Affiliations:** 1 Institute of Environmental and Animal Hygiene and Veterinary Medicine (with Animal Clinic), University Hohenheim, Stuttgart, Germany; 2 Center for Microscopy and Image Analysis, University of Zurich, Zurich, Switzerland; 3 Institute of Veterinary Bacteriology, Vetsuisse Faculty, University of Zurich, Zurich, Switzerland; 4 Laboratory of Vascular Immunology, Division of Internal Medicine, University Hospital Zurich, Zurich, Switzerland

## Abstract

Hemotrophic mycoplasmas (HM) are highly specialized red blood cell parasites that cause infectious anemia in a variety of mammals, including humans. To date, no in vitro cultivation systems for HM have been available, resulting in relatively little information about the pathogenesis of HM infection. In pigs, *Mycoplasma suis*-induced infectious anemia is associated with hemorrhagic diathesis, and coagulation dysfunction. However, intravasal coagulation and subsequent consumption coagulopathy can only partly explain the sequence of events leading to hemorrhagic diathesis manifesting as cyanosis, petechial bleeding, and ecchymosis, and to disseminated coagulation. The involvement of endothelial activation and damage in *M. suis*-associated pathogenesis was investigated using light and electron microscopy, immunohistochemistry, and cell sorting. *M. suis* interacted directly with endothelial cells in vitro and in vivo. Endothelial activation, widespread endothelial damage, and adherence of red blood cells to the endothelium were evident in *M. suis*-infected pigs. These alterations of the endothelium were accompanied by hemorrhage, intravascular coagulation, vascular occlusion, and massive morphological changes within the parenchyma. *M. suis* biofilm-like microcolonies formed on the surface of endothelial cells, and may represent a putative persistence mechanism of *M. suis*. In vitro analysis demonstrated that *M. suis* interacted with the endothelial cytoskeletal protein actin, and induced actin condensation and activation of endothelial cells, as determined by the up-regulation of ICAM, PECAM, E-selectin, and P-selectin. These findings demonstrate an additional cell tropism of HM for endothelial cells and suggest that *M. suis* interferes with the protective function of the endothelium, resulting in hemorrhagic diathesis.

## Introduction

Hemotrophic mycoplasmas (HM) are global etiological agents of infectious anemia in a variety of animals. HM are small erythrocytic parasites that adhere to and invade red blood cells (RBCs) [1-3]. To date, no in vitro cultivation system for HM has been established. In the past few years, reports of zoonotic human HM infections have increased markedly. Strains of *Mycoplasma suis*, *M. haemofelis* and *M. ovis* have been isolated from diseased humans [4-6]. Additionally a novel HM species, termed *Candidatus M. haemohominis,* was recently reported in a human subject and was associated with clinical symptoms of pyrexia and hemolytic anemia [7].

In pigs, acute *M. suis* infection (infectious anemia in pigs, or IAP) manifests as hemolytic anemia and hemorrhagic diathesis accompanied by immune modulation and coagulation dysfunction due to intravasal coagulation and subsequent consumption coagulopathy [8-13]. However, disseminated intravascular coagulation cannot fully account for the clinical signs of acute *M. suis* infection, e.g. cyanosis, petechial bleeding, and ecchymosis. One possibility is that the endothelial cells (ECs) of the vasculature are involved in the pathogenesis of IAP. Electron microscopic studies of RBCs from experimentally-infected pigs revealed massive morphological changes as well as strong aggregation of parasitized and non-parasitized cells. Blood clotting in these animals was attributed to the production of cold and warm autoreactive IgM and IgG antibodies directed against the RBC surface. In addition an acute phase response of the immune system enhanced the formation of blood clots [8,13,14].

To date, several pathogenic bacteria, including *M. mycoides* subsp. *mycoides*, *M. gallisepticum, M. neurolyticum, Bartonella bacilliformis, Rickettsia conorii* and *Bacillus anthracis*, have been described that target and damage the endothelial barrier, or interact closely with EC, triggering inflammatory responses and coagulation processes. In this manner, they are able to interfere with the protective function of ECs through a variety of mechanisms, including invasion and toxin induced endothelial damage, that latter of which could induce hemorrhagic diathesis [15-20].

Based on current evidence, RBCs serve as the exclusive target and host cell of *M. suis*. The goal of the current study was to elucidate potential interactions between *M. suis* and ECs both in vivo and in vitro. Our hypothesis is that the interaction of *M. suis* with ECs, via either adhesion and/or activation, leads to an activated EC phenotype, thereby mediating damage to the vascular endothelium and activation of pro-inflammatory and inflammatory cascades. These direct and indirect interactions induce a systemic immune response resulting in endothelial pathophysiology. We demonstrated that *M. suis* closely interacts with ECs in vivo and in vitro, resulting in endothelial activation and destruction. *M. suis* infection led to endothelial damage, the induction of pro-inflammatory and inflammatory cascades, and immunopathology.

## Materials and methods

### Experimental infections

Experimental *M. suis* infection was performed as described previously [21-23] in accordance with Swiss legislation for animal welfare (Veterinary Office of Zurich, Switzerland; approval 55/2007; 68/2009). The *M. suis* negative health status was confirmed by a quantitative PCR [24] and ELISA [22]. Afterwards, all piglets (*n* = 10) underwent splenectomy [25]. Six pigs were infected intramuscularly with 2 mL of *M. suis*-containing blood from an experimentally infected pig, as described previously (strain 3806; 1 × 10^8^*M. suis*/mL blood [1]), the remaining four pigs were mock-infected (2 mL *M. suis*-negative porcine blood) and served as a negative control. Splenectomy induces an acute clinical manifestation of *M. suis* infection in the used pig model [25,26]. Pigs were scored daily for the following parameters: feeding behavior, body temperature, and clinical signs, as previously described [26]. Briefly, a score of 1 was given for each occurrence of reduced food uptake, fever (> 40°C), lethargy, and pale skin/ear necrosis. Individual scores were then summed to arrive at an overall score for each animal. When a score of 4 was reached (clinical attack), pigs were treated with tetracycline (intramuscularly, 40 mg/kg body weight) and glucose (35 g/L drinking water). In case of recovery failure the pigs were euthanized. *M. suis*-negative pigs were monitored accordingly.

### Cell culture

Primary porcine aortic endothelial cells (PAEC; European Collection of Cell Cultures, Salisbury, UK) were used for the in vitro adhesion assays. The immortalized porcine aortic endothelial cell line PEDSV.15 was used for in vitro activation [27]. PAECs were cultured in Porcine Endothelial Growth Medium (CellMade, Archamps, France) containing penicillin (100 U/mL) and streptomycin (100 mg/mL). PAECs between passages three and six were seeded on cover slips in a 24-well tissue culture plate. PAECs cell culture assays were performed at 37°C and 5% CO_2_.

PEDSV.15 cells (a porcine immortalized aortic cell line) were maintained in DMEM supplemented with 10% FCS, 1 mM sodium pyruvate, 1 mM L-glutamine, non-essential amino acids (1×), and 20 mM HEPES (all from Biochrom, Berlin, Germany) [28]. PEDSV.15 cell culture assays were performed at 37°C and 5% CO_2_ in 1.5 mL of DMEM in 12-well tissue culture plates.

### Purification of *M. suis*

*M. suis* was purified from the plasma of infected pigs as described previously, with slight modifications [22,23]. Briefly, sodium citrate anti-coagulated blood was subjected to centrifugation for 5 min at 300 × *g* to sediment the erythrocytes. Plasma was removed and subjected to centrifugation at 20 000 × *g* for 1 h at room temperature (RT) (Hettich Rotixa/AP; Hettich, Tuttlingen, Germany). The resulting pellet was washed twice and then resuspended in phosphate buffered saline (PBS, Biochrom). *M. suis* was quantified by quantitative LightCycler (LC) PCR analysis [24]. As a negative control, blood from non-infected pigs was prepared using the same procedure (negative control preparation).

### Harvesting of blood vessels and parenchyma (heart and liver)

Blood vessels (abdominal aorta and veins) and parenchyma (heart and liver) were collected from euthanized *M. suis*-infected and control pigs to examine the effect of *M. suis* on the ECs. Blood vessels and parenchyma were immediately fixed in 4% phosphate-buffered formaldehyde (FA) for 24 h. Other tissues (mesenteric lymph nodes, liver, kidney, duodenum, jejunum, ileum, colon, pancreas, and spleen) were immediately fixed in methanol/glacial acid (2:1 for 24 h) or in Bouin’s fluid, and then embedded in paraffin according to standard procedures.

### Light microscopy and scanning electron microscopy of aortic vessels

FA-fixed blood vessels and parenchyma were post-fixed in 2.5% phosphate-buffered glutaraldehyde (GA) for 24 h and then stored in 0.1 M cacodylate buffer until further processing. Strips 2 cm in length (long axis) of the fixed blood vessels were cut in half for macroscopic documentation under a Leica Z16 APO light microscope (Leica Microsystems, Heerbrugg, Switzerland). After microscopy, tissues were post-fixed for 1 h at RT in 1% osmium tetroxide (Fluka Chemie, Buchs, Switzerland) in 0.1 M cacodylate buffer, dehydrated through a graded ethanol series, and then subjected to critical point drying (BAL-TEC CPD 030, Critical Point Dryer, Balzers, Liechtenstein). Finally, the samples were sputter-coated with 12 nm of platinum using the BAL-TEC MED 020 coating system, mounted on an aluminum stub, and then analyzed on a Zeiss Supra 50 VP (Oberkochen, Germany) scanning electron microscope.

### Immunohistochemical staining of aortic vessels and parenchyma

For immunohistochemistry, the fixed and paraffin embedded tissues were cut into sections (5 μm thick) and mounted on Superfrost® glass slides. After deparaffinization and antigen retrieval in a microwave oven in 10 mM sodium citrate buffer at pH 6.0 (3 × 5 min at 700 W), sections were subjected to immunohistochemistry using an *M. suis*-specific rabbit polyclonal antibody directed against α-enolase (1:200 in PBS) [29]. Briefly, sections were treated with 1% hydrogen peroxide in double-distilled water for 10 min at RT to block endogenous peroxidase activity followed by 10% normal goat serum (Dako, Hamburg, Germany) for 30 min at RT to prevent non-specific protein binding. This was followed by incubation with the primary antibody at 5°C overnight in a humid chamber. The next day, sections were incubated with biotinylated goat anti-rabbit IgG (1:100 in PBS; Dako) for 30 min at RT. Antibodies were detected using a Strept-ABC kit (Dako) according to manufacturer’s instructions. Each incubation step was followed by 3 × 5-min rinses with PBS. The reaction product was visualized with 3,3′-diaminobenzidine-hydrogen-peroxide reagent (DAB) (Biotrend Chemicals, Köln, Germany). Finally, sections were counterstained with Mayer’s hematoxylin, dehydrated, cleared with xylene, and then mounted in Entellan (Merck, Darmstadt, Germany). The samples were analyzed using a bright field light microscope (DMRBE, Leica, Bensheim, Germany) equipped with a video camera (ProgRes, Kontron Instruments, Watford, UK). Immunohistochemical controls were performed by (1) replacing the primary antibody with non-immune serum, (2) omitting the secondary antibody, and finally (3) incubation with DAB solution alone to ensure specificity of staining.

### Transmission electron microscopy of aortic vessels and parenchyma

Formaldehyde-fixed blood vessels and parenchyma were cut into 1–2 mm segments and post-fixed in 2.5% phosphate buffered GA solution for 24 h. Samples were washed in 0.05 M cacodylate buffer and stored at 4°C until further processing. Following post fixation in 2% osmium tetroxide in cacodylate buffer, sections were washed once in 0.05 M cacodylate buffer. Samples were dehydrated in increasing concentrations of ethanol (70–100%) and then infiltrated by increasing concentrations (33 and 50%) of Epon 812 (Fluka Chemie) in ethanol (100%) for 1 h each. After incubation in a mixture of 75% Epon and 25% ethanol overnight, samples were infiltrated with fresh pure Epon for 2–3 h. Specimens were embedded in fresh Epon in flat silicone rubber molds and hardened for 12 h at 60°C. After preparation of ultrathin sections using an Ultracut E microtome (Reichert-Jung, Vienna, Austria), sections were placed on copper grids (Plano GmbH, Wetzlar, Germany), contrasted consecutively with 4% uranyl acetate (Fluka Chemie) and lead citrate as described previously (Reynold 1963), and then analyzed with a Philips CM100 transmission electron microscope equipped with a Gatan Orius CCD camera (Gatan, Munich, Germany).

### Immunogold staining of *M. suis* in aortic tissue

FA-fixed blood vessels were cut into 5–8 mm segments, equilibrated in 30% sucrose and then embedded in tissue-Tek (Sakura Finetek, Staufen, Germany) at −40°C. Horizontal segments (40 μm thick) were sectioned using a Kryostat HYRAX C 60 (Zeiss, Jena, Germany) to view the subcellular matrix underneath the endothelium. Sections were permeabilized with 0.2% Triton X-100 (Sigma-Aldrich, Buchs, Switzerland) for 1 h at RT and then incubated for 24 h with a monoclonal antibody against the *M. suis* surface protein, MSG1 (10 μg/mL). Sections were then washed three times for 20 min each with PBS and then incubated with 1.2 nm colloidal gold particles conjugated to goat anti-mouse IgG (1:1000) [30] for 1 h at RT. Sections were washed three times for 15 min each with PBS and then post-fixed for 30 min in 2.5% GA. After GA fixation, sections were washed twice with distilled water for 20 min. Silver enhancement was performed using the silver enhancement kit (BBI International, Cardiff, United Kingdom). After post-fixation with 2% osmium tetroxide in PBS, sections were prepared for EM as described for aortic vessels and parenchyma.

### In vitro adhesion of *M. suis* to PAECs

PAECs on cover slips were incubated with purified *M. suis* (1 × 10^4^*M. suis*/mL; 100 μL/well) or the negative control purification preparation (100 μL/well) for different time periods (5 min to 5 days). After washing twice with PBS, cells were fixed in 2.5% phosphate buffered FA for 2 h at 4°C. After washing again with PBS, PAECs were permeabilized with 0.2% Triton X-100 (Sigma-Aldrich) for 2 min at RT. Cells were washed with PBS and non-reacted aldehydes were blocked with 0.1 M glycine (Carl Roth, Karlsruhe, Germany) in PBS for 20 min. Non-specific binding of antibodies was reduced by incubation of samples in blocking buffer (3% BSA in PBS) for 30 min. Adhesion of *M. suis* to PAECs was visualized by staining with rabbit anti-HspA1 serum (1:100) [31] for 1 h followed by TRITC-conjugated goat anti-rabbit IgG (Sigma-Aldrich) for 1 h. Filamentous actin in the PAECs was visualized using FITC-phalloidin (1:80 in PBS; Invitrogen, Basel, Switzerland) and the nucleus was counterstained with DAPI (250 ng/mL). The samples were analyzed on a Leica SP2 confocal laser scanning microscope (CLSM, Leica Microsystems).

### FACS analysis of adhesion molecule expression upon interaction with *M. suis*

To analyze the activation of endothelial cells by *M. suis*, PEDSV.15 cells at 60% confluence were incubated in duplicate with purified *M. suis* (1 × 10^4^ cells/mL; 0.5 mL in PBS) or the negative control purification preparation. Cells were harvested and fixed in 2.5% FA after 2 h and 4 h for the analysis of E (CD62E)- and P (CD62P)-selectin expression, respectively, and after 24 h, for the analysis of intercellular adhesion molecule 1 (ICAM-1; CD54) and platelet endothelial cell adhesion molecule 1 (PECAM-1; CD31) expression [32-34]. These time points corresponded to the maximum up-regulation of activation markers determined with the positive control, i.e. tumor necrosis factor α (TNF-α) and lipopolysaccharide (LPS; Sigma). Fixed cells were incubated with 100 μL of blocking buffer (10% BSA in PBS) for 30 min at RT, followed by specific antibodies directed against ICAM-1 (clone 19C7; a kind gift from D. Haskard, Imperial College London, UK), PECAM-1 (LCI-4; AbD Serotec, Oxford, UK), P-selectin (clone 12C5; D. Haskard) and E-selectin (clone 1.2B6; AbD Serotec) as previously described [35,36]. FITC-conjugated goat anti-mouse IgG (1:1000; Sigma-Aldrich) was used as a secondary antibody. Samples were analyzed using a FACSCanto II cell sorting system (BD Biosciences, Allschwil, Switzerland). Experiments were performed three times.

SPSS statistical software was used to test differences in the expression of cell surface markers between groups. Differences were calculated using the unpaired Wilcoxon test and a *P* value ≤ 0.05 was considered statistically significant.

## Results

### Endothelial cell alteration and activation in *M. suis*-infected pigs

**Figure 1:**
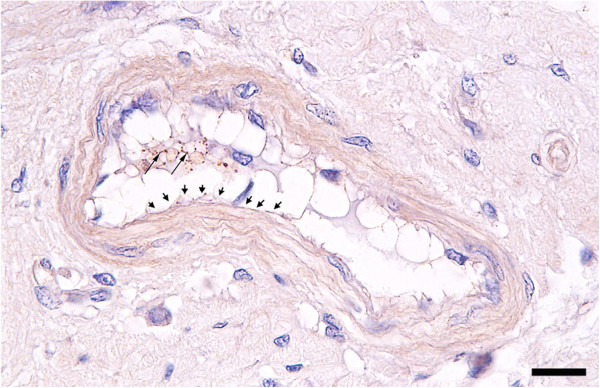
**Degeneration of capillary endothelial cells in *M. suis *-infected pigs.** Representative small intestinal venule of an *M. suis*-infected pig (histological analysis). Massive cytoplasmic swelling of ECs, accompanied by progressive cellular necrosis and detachment of ECs from the underlying basement membrane, is evident. Arrowheads indicate areas of cellular denudation of the luminal surface; Arrows indicate immunoreactive *M. suis* cells. Scale bar = 20 μm.

**Figure 2:**
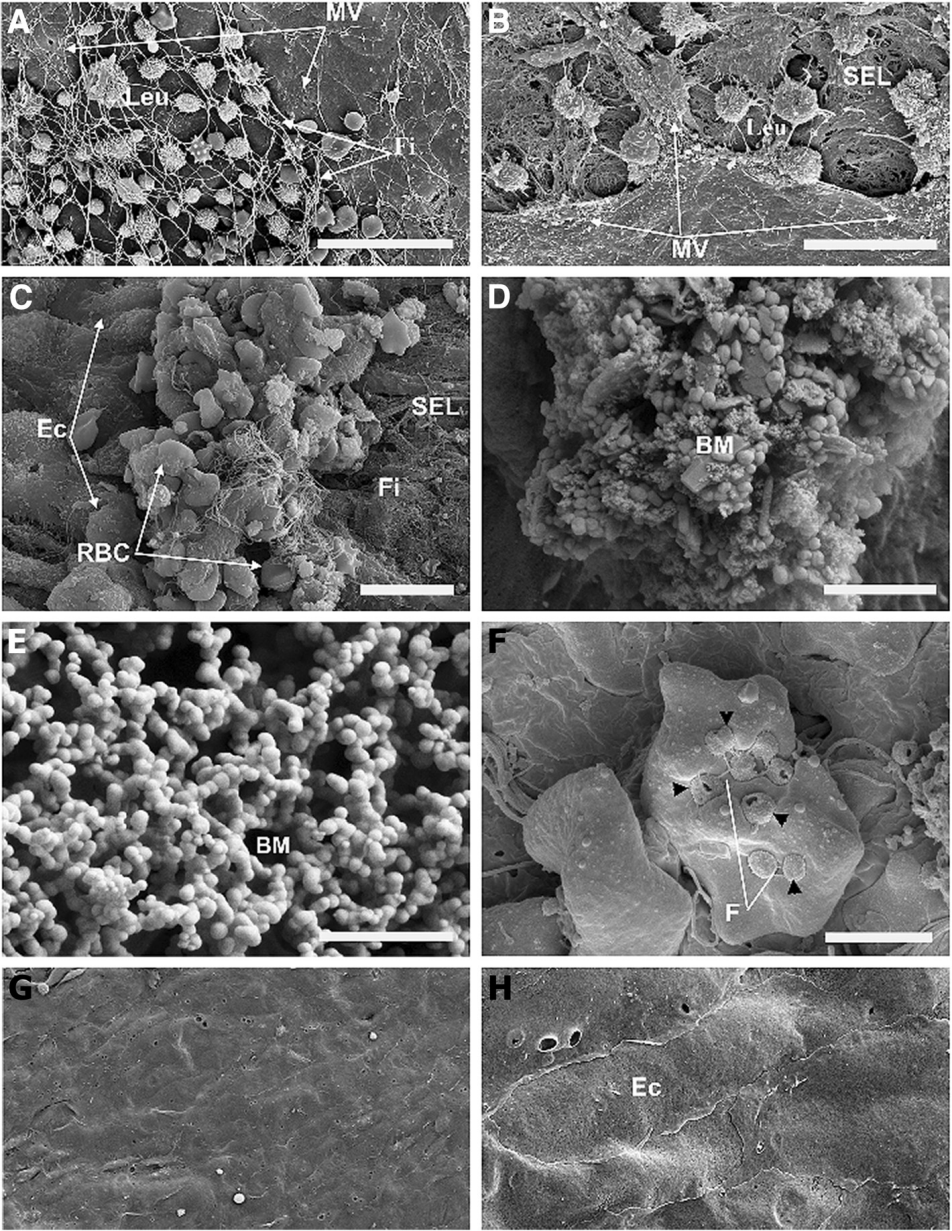
**Endothelial denudation and microvilli and microcolony formation due to *M. suis *infection. A**. Aortic vessel of an infected pig with adherent blood cells and fibrin fibers attached to ECs. Scale bar = 20 μm; **B**. Aortic vessel from an infected pig showing activated ECs (microvilli formation) and detached ECs exposing the subendothelial layer (SEL). Leukocytes are attached to the ECs and the subcellular matrix. Scale bar = 10 μm; **C**. Vascular wall covered with blood clots. Scale bar = 10 μm; **D**. Bacterial microcolonies (BMs) attached to the vascular wall. Scale bar = 2 μm; **E**. *M. suis* coccoids of less than 100 nm. Scale bar = 2 μm; **F**. *M. suis* cells attached to RBCs and interconnected via small fibrils. Scale bar = 2 μm; **G**. Luminal surface of an aortic vessel from a healthy pig showing a confluent layer of ECs (overview). Scale bar = 40 μm; **H**. Aortic vessel from a healthy pig showing a confluent layer of ECs. Scale bar = 10 μm. Abbreviations: BM, bacterial microcolony; EC, endothelial cell; Fi, fibrinogen fibers; Leu, leukocytes; MV, microvilli; RBCs, red blood cells; F, small fibrils; SEL, subendothelial layer; and VW, vessel wall.

Microscopic analysis of the vascular endothelium of *M. suis*-infected pigs revealed marked alterations of the luminal surface of the abdominal aortic vessel, which appeared rough and covered in reddish spots. An additional file shows this in more detail (see Additional file 1a). These red dots indicated aggregated blood cells and perforation of the vessel walls. By comparison, the endothelial surface of the abdominal aortic vessel in healthy pigs exhibited a smooth and even surface structure as shown in an additional file (see Additional file 1b). Histological and immunohistochemical analysis of the organs of *M. suis*-infected pigs (duodenum, jejunum, ileum, colon tissue, heart, lung, liver, mesenteric lymph nodes, and spleen) revealed massive alterations in tissue structure, particularly in the vascular system (Figures 1 and 2). The Additional files 1a and b shows this in more detail (Additional file 1a and b). Infection with *M. suis* also resulted in injury to the ECs lining the post-capillary venules and small veins. As shown in Figure 1, early signs of degeneration, i.e. massive cellular protrusions into the vessel lumen, were accompanied by acute cytoplasmic swelling. Desquamation, necrosis and detachment of ECs from the underlying basement membrane were observed, resulting in progressive cellular denudation of the luminal surface. Numerous *M. suis* cells could be detected within the detached endothelium (Figure 1).

Scanning electron microscopy (SEM) of the aortic vessel of *M. suis*-infected pigs revealed areas of endothelial denudation leading to an exposed sub-endothelial matrix. In the endothelial gaps, there were large numbers of leukocytes embedded within a fibrin network. The remaining ECs were covered with numerous microvilli, indicating an inflammatory response (Figure 2a and 2b). An additional file shows this in more detail (see Additional file 2). Areas with adherent blood cells, which had appeared as red dots by light microscopy, where identified as arterial thrombi by SEM. These arterial thrombi were characterized by the presence of RBCs and leukocytes embedded in a network of fibrin fibers (Figure 2c). In summary, the observed alterations provided evidence of hemorrhages due to *M. suis* induced endothelial damages.

SEM also revealed *M. suis* aggregates of approximately 5–50 μm in diameter attached to the surface of the vessel walls. These micro-colonies were characterized by round or elongated cross-linked cells of 200–400 nm in diameter embedded in a three-dimensional granular matrix (Figure 2d). In addition, smaller coccoids (less than 100 nm in size) localized to the surface of the larger bacterial cells (Figure 2e). In some cases, *M. suis* cells on RBCs were interconnected via small fibrils (Figure 2f). In contrast to aortic vessels from *M. suis*-infected pigs, the aortic vessels of healthy pigs showed no evidence of endothelial cell damage. Confluent layers of spindle-shaped ECs (5–8 μm in width and 10–25 μm in length) arranged in parallel and with no attached blood cells or bacteria were observed in non-infected pigs (Figure 2g and 2h).

**Figure 3:**
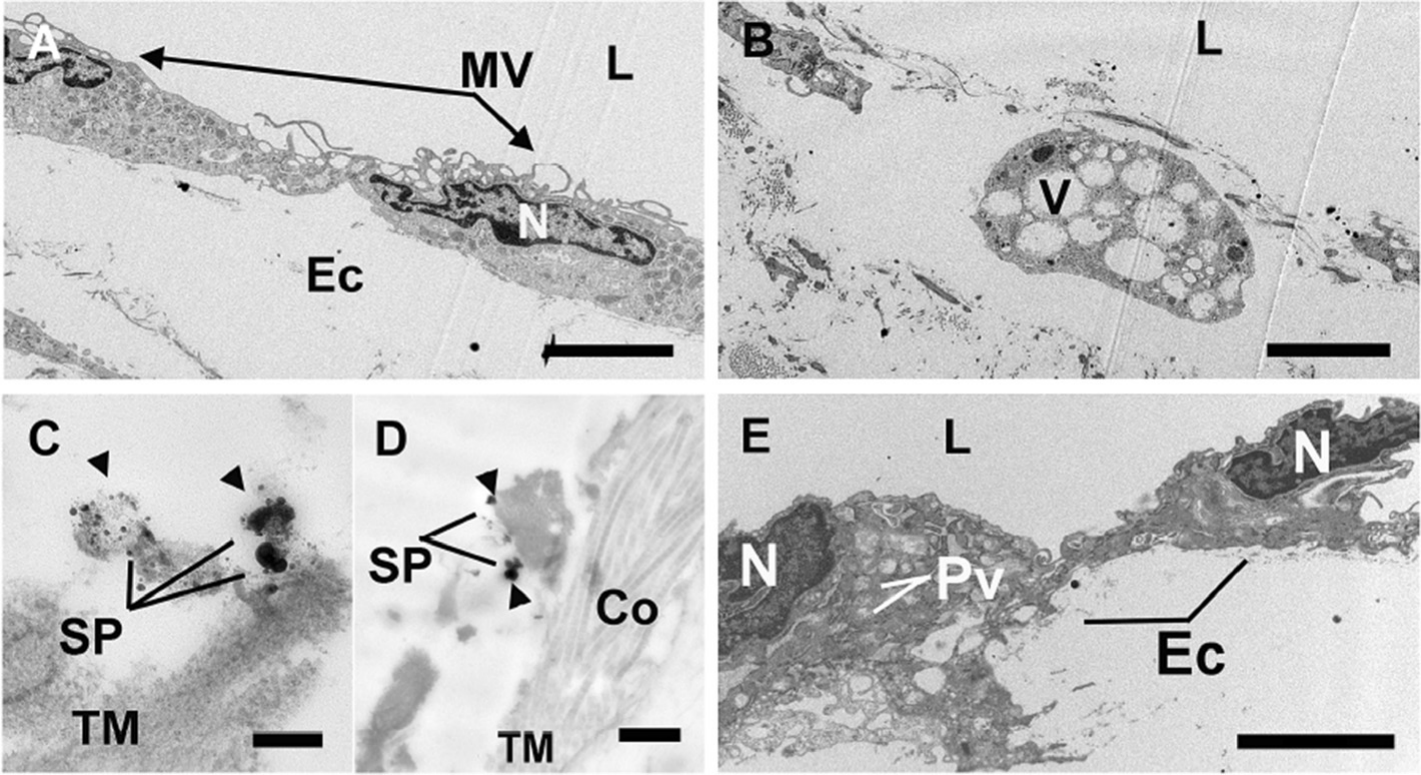
***M. suis *infection induces endothelial cell detachment and activation.** Transmission electron micrographs of aortic sections. **A**. ECs (black arrows) of pigs infected with *M. suis* (black arrowheads) are characterized by extensive microvilli (MV) formation. Scale bar = 4 μm; **B**. Absence of ECs/EC detachment and remaining vacuolar cells. Scale bar = 4 μm; **C**, **D**. *M. suis* was detected in the aortic sub-cellular tissue by nanogold silver intensification staining. Scale bar = 0.2 μm (**C**); Scale bar = 0.5 μm (**D**); **E**. Endothelial cells (ECs) of uninfected control pigs showing normal ultrastructure. Scale bar = 4 μm. Abbreviations: Co, collagen; L, lumen; PV, plasmalemma vesicle; N, nucleus; SP, silver precipitates; RM, tunica media; and VC, vacuolar cell.

Ultrathin sections of the abdominal aorta were further analyzed for EC activation and detachment by transmission electron microscopy (TEM). Microvilli formation was evident on the ECs of the abdominal aorta of *M. suis*-infected pigs (Figure 3a). In some ultrathin sections, no ECs were detected at all, a finding consistent with the SEM analysis of the aorta of the same pig (Figure 3b). *M. suis* was detected in the subcellular aortic tissue following staining with an anti-MSG1 monoclonal antibody and subsequent nanogold silver intensification (Figure 3c, d). *M. suis* cells appeared to be attached to subcellular matrix structures resembling collagen. As with the results of SEM, the vascular wall of healthy control pigs was characterized by a continuous layer of ECs with no alterations in ultrastructure (Figure 3e).

### Intravascular coagulation and morphological changes in the parenchyma in *M. suis*-infected pigs

To further examine *M. suis*-induced morphological changes, endothelial morphology and intravascular coagulation in the organ capillaries of the liver and heart parenchyma were analyzed by TEM.

**Figure 4:**
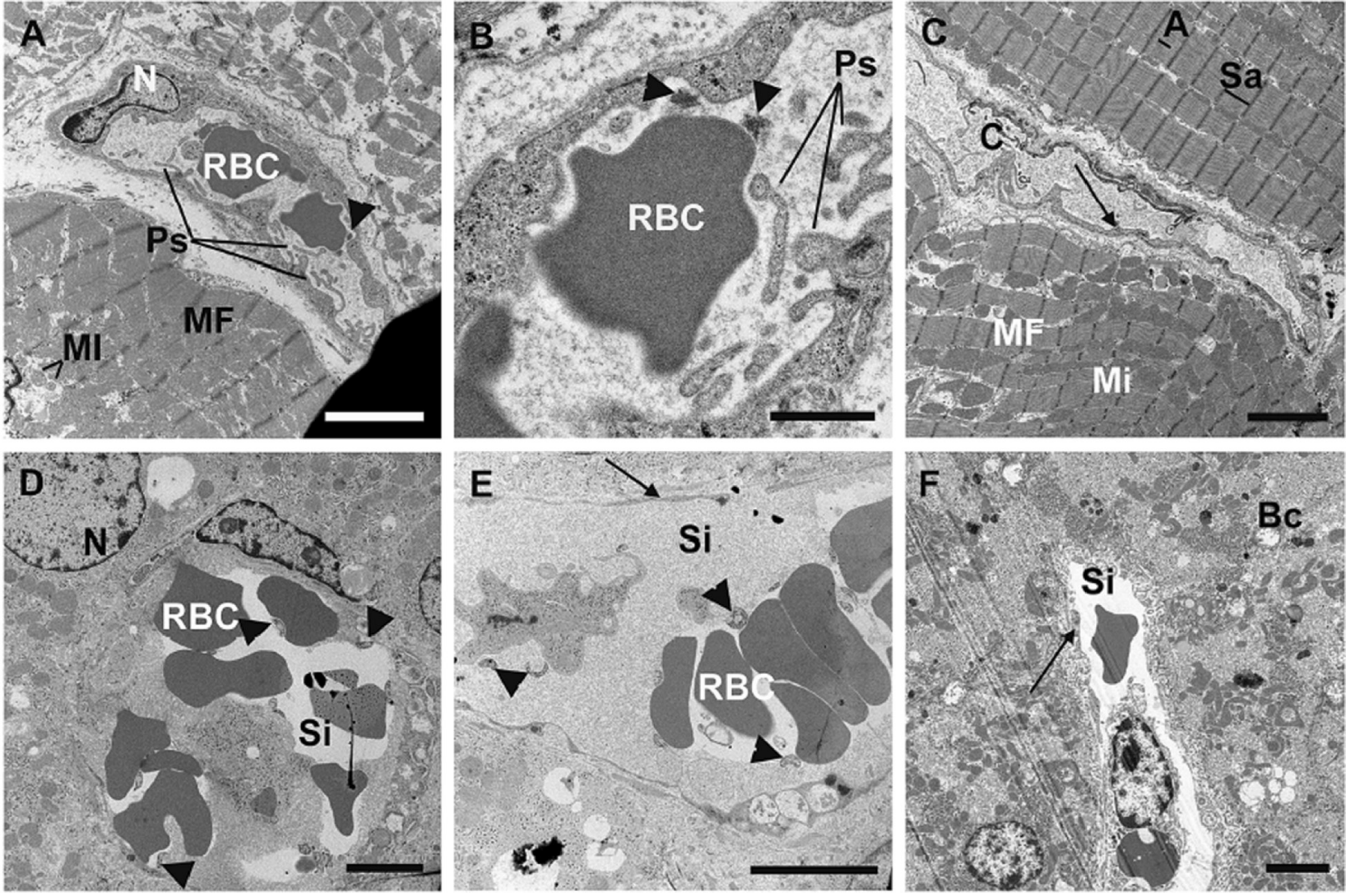
**Intravascular coagulation and morphological changes in *M. suis *infected pigs.** Transmission electron micrographs of porcine liver and heart preparations. **A**, **B**. Heart biopsies from an *M. suis*-infected pig with unorganized myofibrils (MF)*. M. suis* cells are indicated by a black arrowhead and thin endothelial cells of capillaries (c) and sinusoids (Si) with a black arrow. Scale bar = 4 μm (A); scale bar = 4 μm (B); **C**. Heart biopsy from an uninfected control pig. Myofibrils exhibit the typical banding pattern with alternating A and I bands. Scale bar = 4 μm. **D**, **E**. Liver preparations from an *M. suis*-infected pig with red blood cell (RBC) aggregates adherent to endothelial cells (ECs). Scale bar = 4 μm (D); scale bar = 1 μm (E); **F**. Liver preparation from an uninfected control pig. Scale bar = 4 μm. Abbreviations: Mi, mitochondria; N, nucleus; Ps, pseudopodia; RBC, red blood cell; ER, endoplasmic reticulum; V, intracellular vacuoles; and Bc, bile canaliculi.

The ECs of the heart capillaries of *M. suis*-infected pigs were irregular in shape with localized swelling and finger-like pseudopodia (Figure 4a, b). *M. suis*-infected RBCs were adherent to the endothelium and surrounded by endothelial pseudopodia.

Morphological changes in the heart parenchyma of infected pigs were also observed. In cardiac muscle cells (Figure 4a), myofibrils were disarranged and the number of fibrils was reduced. Mitochondria were markedly reduced in number and appeared to be randomly distributed in the heart tissue. Sarcomere atrophy was evident and the structures inconsistent in many parts of the tissue (Figure 4a).

In control pigs, by comparison, there was a regular pattern of ECs lining the cardiac vessel wall. No RBCs were attached to the vessel wall and no *M. suis* particles were detected (Figure 4c). The morphology of the cardiac muscle of control pigs was normal, with alternating I and A bands. Sarcomeres were arranged in parallel and myofibrils were separated by the normal striped arrangement of mitochondria.

Intravascular coagulation was detected in the microvascular channels (sinusoids) of the liver of *M. suis*-infected pigs. Aggregated RBCs attached to the vascular wall appeared to block the sinusoids (Figure 4d, e). In contrast, plasma in the sinusoids of control pigs had a homogenous appearance and RBCs were distinct and separated from the vessel wall (Figure 4f). Liver cells (hepatocytes) from *M. suis*-positive pigs (Figure 4d, e) and control pigs (Figure 4f) had a normal polygonal structure and showed no signs of morphological changes. Bile canaliculi, the thin tubes that collect bile secreted by hepatocytes, were also displayed.

### *M. suis* interaction with and activation of porcine aortic endothelial cells in vitro

**Figure 5:**
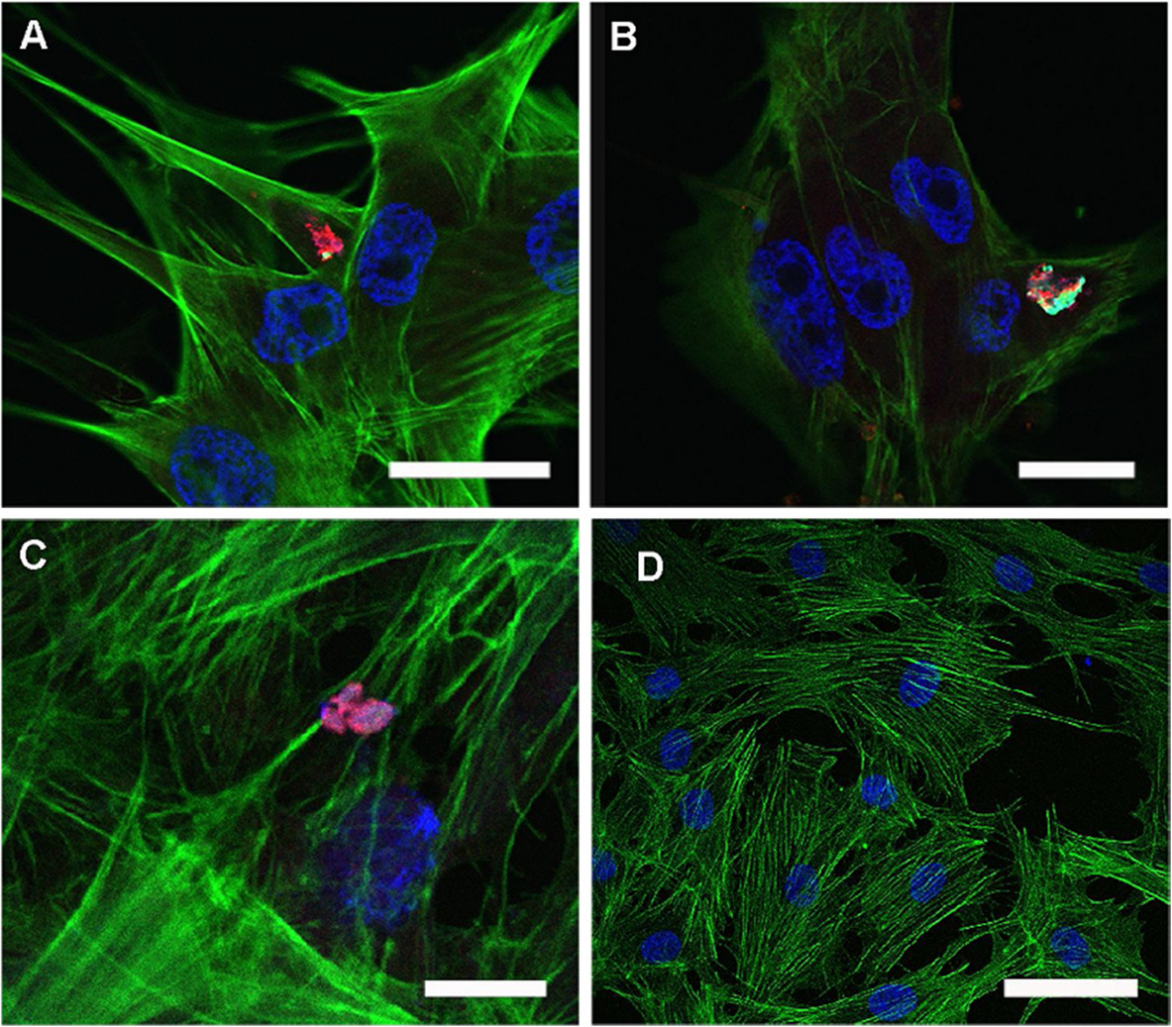
**Interaction of *M. suis *with porcine endothelial cells induces actin condensation in vitro.** Confocal laser scanning micrographs of PAECs incubated with *M. suis* (1 × 10^4^ cells/mL). Cytoskeletal actin was stained with FITC-phalloidin (green); *M. suis* cells were stained with anti-HspA1 antiserum and TRITC-labeled secondary antibodies (red). Nuclei and bacteria were counterstained with DAPI (blue). The image represents an overlay of all fluorescent channels. (**A**) 90 min post-infection (pi); scale bar = 40 μm; (**B**) 6 hours pi; scale bar = 20 μm; (**C**) 4 days pi; scale bar = 10 μm; (**D**) Pre-infection; scale bar = 50 μm.

To investigate the interaction of *M. suis* with ECs in vitro, PAECs were infected with purified *M. suis* and then analyzed by confocal laser scanning microscopy following immunohistochemical staining to differentiate the cytoskeleton, cell nucleus, and *M. suis. M. suis* cells were detected as aggregates on the surface of PAECs as early as 90 min post-infection. Actin condensation was observed at the sites of bacterial cell attachment (Figure 5a–c) and always co-localized to sites of bacterial aggregates. Propagation of *M. suis* on the surface of PAECs was not observed over the course of this experiment. As a control, no *M. suis* cells were detected on PAECs incubated with the negative control preparation (Figure 5d).

To assess whether the presence of *M. suis* cells in the medium in absence of immune components activates ECs*,* PEDSV.15 cells were infected with purified *M. suis*. Expression of different cell adhesion molecules (CAMs), i.e. ICAM-1 (CD54), PECAM-1 (CD31), P-selectin (CD62P) and E-selectin (CD62E) was used as activation marker. The CAMs expression levels (number of CAMs expressing PEDSV.15 cells) were determined using FACS analysis and CAM-specific monoclonal antibodies. PEDSV.15 cells were responsive for activation as shown by the induction of CAMs upregulation after TNF-α and LPS stimulation. TNF-α induces a 2.3 fold more CD54, a 2.0-fold more E-selectin (CD62E), a 15.8-fold more P-selectin CD62P, and 2.1-fold PECAM-1 (CD31) expression when compared with the negative control (cell culture medium). LPS induces a 17.5 fold more CD54, a 6.9-fold more E-selectin (CD62E), a 14-fold more P-selectin CD62P, and 2.1-fold PECAM-1 (CD31) expression when compared with the negative control (cell culture medium).

**Figure 6:**
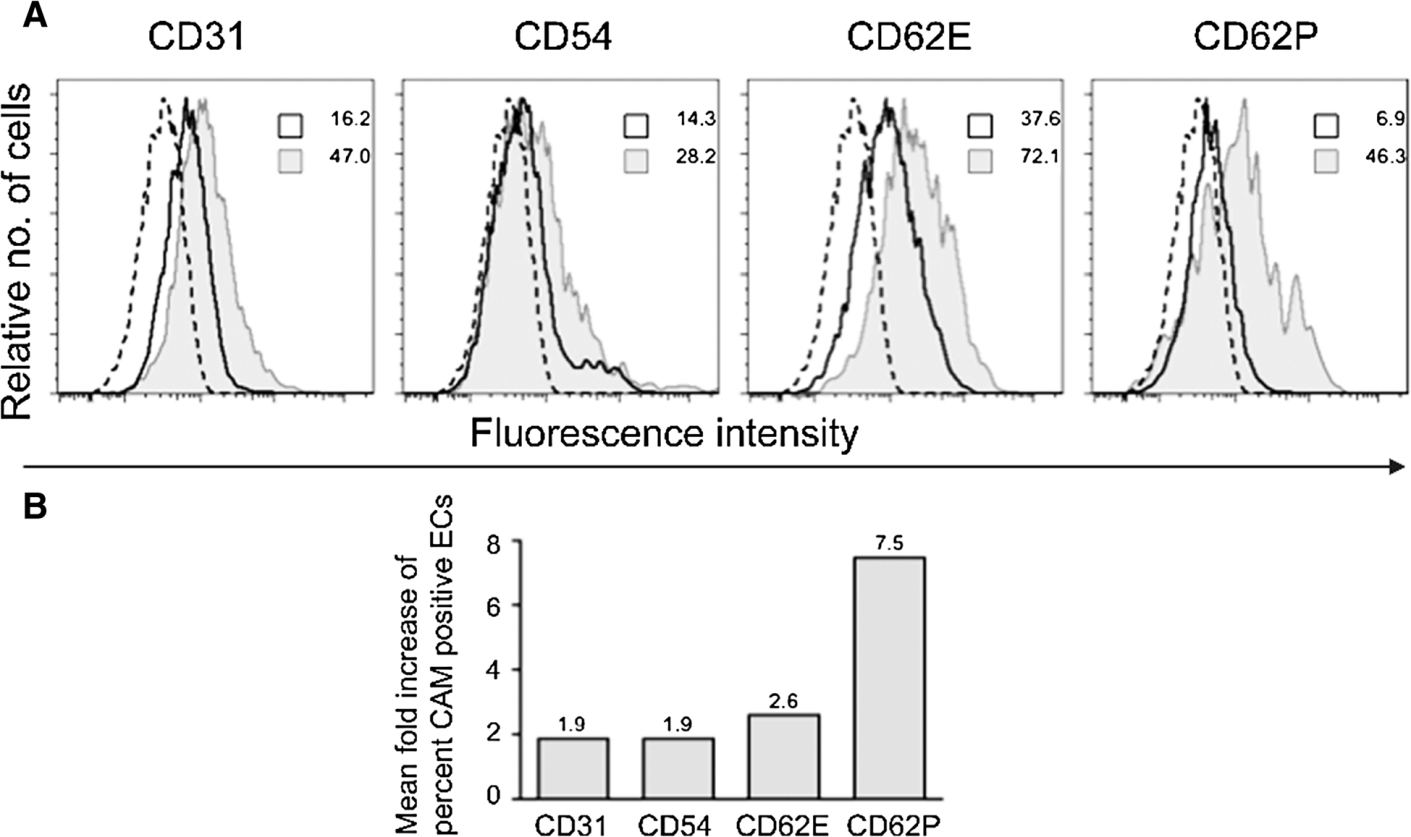
**CAM expression in *M. suis *infected endothelial cells.** The expression profiles of adhesion molecules on endothelial cells were analyzed using a FACSCanto II cell sorting system. **A**. Dashed lines represent the isotype control; solid lines represent cells incubated with the negative control preparation; grey histograms represent PEDSV.15 cells incubated with *M. suis* (1 × 10^4^ cells/mL). **B**. Percent increase in expression ICAM-1 (CD54), PECAM-1 (CD31), E (CD62E)- and P (CD62P)-selectin in ECs infected with *M. suis*.

Overall, there was a significant increase in CAMs expressing PEDSV.15 cells upon infection with *M. suis* (*P* ≤ 0.05*)*. As shown in Figure 6*M. suis*-stimulated PEDSV-15 showed a significant increase in CAM cell-surface expression compared with that in the negative control preparation *M. suis*-incubated PEDSV-15 expressed on average 1.9-fold more ICAM-1 (CD54, *P* = 0.028), 2.6-fold more E-selectin (CD62E, *P* = 0.043), 7.5-fold more P-selectin (CD62P, *P* = 0.028) and 1.9-fold more PECAM-1 (CD31, *P* = 0.043) compared with negative control preparation.

## Discussion

Hemolytic anemia as a result of acute *M. suis* infection is accompanied by coagulopathy, vascular thrombosis, and hemorrhaging of the skin and organs; symptoms that are indicative of the involvement of vascular ECs in the pathogenesis of acute *M. suis* infection [9,11,12]. However, the involvement of ECs in *M. suis* pathogenesis has received little attention to date because consumption coagulopathy is considered to be the primary cause of hemorrhagic diathesis [12]. Our own recent clinical observations of acute IAP with a fatal outcome [1] led to the hypothesis that interactions with ECs contribute to IAP pathogenesis. Therefore, the aim of the present study was to investigate the interaction between *M. suis* and ECs, and the resulting endothelial disruption and damage to the vasculature.

SEM and TEM analyses clearly demonstrated marked abnormalities in the endothelium of blood vessels in *M. suis*-infected pigs. Blood vessels exhibited detached ECs over a considerable portion of the endothelium. In some ultrathin sections, EC were completely missing and the subcellular matrix was exposed. This likely represents the mechanism by which *M. suis* gained access to the sub-endothelial matrix, where it was detected by TEM. However, the extent of endothelial denudation made the microscopic detection of intracellular *M. suis* in ECs unfeasible. An analogy can be made with certain *Bartonella* species, which are also undetectable intracellularly in ECs, even though pathogenesis induced by these species was shown to involve ECs [37]. Additionally, the abnormalities reported herein are typical signs of activated ECs seen in several other diseases, i.e. sickle cell anemia [38,39], and are highly indicative of dysfunctional activation of the endothelium in IAP.

The formation of microvilli-like membranes on ECs was observed in *M. suis*-infected animals, which is also characteristic of an activated phenotype, and could serve to increase the frequency of adhesion of *M. suis*-infected RBCs, as observed in micro vessels from patients infected with *P. falciparum*[40]. The increased adhesion of *M. suis*-infected RBCs to the endothelium might be further enhanced by *M. suis*-induced RBC changes, some of which could trigger a variety of pathologies such as vasoocclusion, endothelial activation, and initiation of the coagulation cascade [41]. The extent of RBC adhesion seen in the liver sinusoids, as well as in heart capillaries, might be a result of a combination of activated ECs with microvilli, altered RBC membranes, and an increased adhesiveness caused by pseudopodia. Similar pathologic patterns are observed in *P. falciparum* infections [42].

Numerous arterial thrombi, composed of leukocytes, RBCs, platelets and a network of fibrin fibers, were attached to ECs next to sites of *M. suis*-induced endothelial injury. This fibrin network acts as a provisional matrix during inflammation and wound healing [43]. Fibrin and fibrinogen metabolites bind to activated platelets and also to vascular EC, thereby activating both cell types [43,44]. Activated ECs facilitate the translocation of phagocytes into the injured tissue to eliminate bacteria via phagocytosis [45]. Furthermore, in diabetes mellitus and sickle cell anemia, fibrinogen enhances adhesion of pathological RBCs to ECs, and this is associated with a high incidence of thrombosis [46,47].

The detection of *M. suis* in direct contact with ECs and attached to the endothelium is an entirely new finding in HM research. To date, HM has been reported to attach and invade a unique host cell, the RBC [1-3]. In the current study, *M. suis* was attached to ECs either as a single cell or as microcolonies ranging in diameter from 30–50 μm. This direct interaction of *M. suis* with ECs could trigger the EC destruction and exposure of the subendothelial matrix observed in the *M. suis*-infected pigs. This would be consistent with other pathogenic *Mycoplasma* species, such as the large colony strains of *M. mycoides* subsp. *mycoides*, which are able to adhere to and destroy caprine ECs, thereby exposing subendothelial collagen and causing vasculitis and coagulation disturbances [18,20].

The *M. suis* biofilm-like structures we observed were homogenous aggregates of cross-linked 200–400 nm *M. suis* cells that formed a three-dimensional network. Occasionally, microcolonies appeared more compact and the bacteria were embedded in a granular matrix. *M. suis* microcolonies attached to the vascular wall and resembling biofilms have been described for other mycoplasmas [48]. Within these microcolonies, smaller coccoid cells less than 100 nm in diameter were observed on the surface of the larger cells. These *M. suis* cells probably originated from a budding-like replication mechanism, similar to that proposed for HM, as well as for other mycoplasmas, and they resembled propagating *M. suis* cells on RBC [1,49,50]. These results strongly suggested that *M. suis* is capable of propagating on ECs.

One of the more novel and more interesting findings of this study was the formation of small fibrils interconnecting several *M. suis* cells on the surface of RBCs and attached to the vascular wall. *M. suis* adhere to host RBCs via fibrils [50,51]. In the current study, the interconnected *M. suis* cells were observed only on the surface of RBCs attached to the endothelium Further studies are needed to better characterize the composition and function of *M. suis* fibrils [52].

In vitro analysis using porcine endothelial cells (PAEC and PEDSV.15 cells) provided further insight into the mechanism of interaction between *M. suis* and ECs. A considerable number of *M. suis* cells were attached to the PAEC surface, and actin condensation was evident at the attachment sites. Indications for an interaction between *M. suis* and porcine actin have been found in a previous study since *M. suis*-induced autoreactive IgG antibodies also bind to actin [8,53]. In the present study, we could document by microscopic analysis that *M. suis* interacts with cytoskeletal actin of endothelial cells leading to cytoskeletal rearrangements in the EC. Various invasive bacteria, such as *Yersinia enterocolitica* and *Listeria monocytogenes,* trigger rearrangement of the actin cytoskeleton to facilitate their uptake into eukaryotic cells [54,55]. Whether *M. suis* is internalized by a similar mechanism remains to be clarified in further studies. Analysis of the *M. suis* genome revealed that, similar to other *Mycoplasma* species, *M. suis* lack any of the genes required for *de novo* nucleotide biosynthesis [56]. Thus, establishing itself in a new niche in a nucleated cell would be of critical importance for *M. suis* in terms of acquiring a source of nucleotides for proliferation. Despite demonstrating a clear and close interaction between *M. suis* and ECs, definitive proof of the intracellular localization of *M. suis* in ECs in vivo could not be demonstrated due to the extensive cytopathological effects of *M. suis* (data not shown). Damaged ECs most likely detached from the endothelium and, thus, were not available for microscopic analysis of intracellular *M. suis*. Additional studies of sections from infected blood vessels at an early phase of infection may provide the necessary evidence to support this hypothesis.

The activation of ECs by *M. suis* was demonstrated in vitro by the significant increase of ICAM-1, PECAM-1, E-selectin and P-selectin expressing cells, all of which are important markers of EC activation. Endothelial E-selectin and P-selectin are up-regulated by inflammation and mediate leukocyte capture and rolling on the endothelium [57]. The up-regulation of E-selectin is associated with organ dysfunction and septic shock [58], both of which are seen in acute IAP. The time point of a maximum upregulation of activation markers by LPS and TNF-alpha might not represent the time point of a maximum upregulation by *M. suis*. This could explain the relatively smaller but significant difference between *M. suis* and negative control in case of E-selectin and PECAM-1. ICAM-1 is involved in leukocyte rolling and arrest on endothelial cells [57], as well as the movement of neutrophils and monocytes on the endothelium [59]. Recently, ICAM-1 was identified as the receptor for rhinovirus [60]. PECAM-1 is involved in the removal of apoptotic neutrophils from the body and makes up a large proportion of the endothelial cell intercellular junction [61,62], where it mediates transendothelial migration via homotypic binding to PECAM-1 on leukocytes [63]. In summary, these in vitro findings, including the up-regulation of endothelial adhesion receptors, together with the observed structural changes of the endothelial layer in vivo, i.e. microvilli- and gap-formation, demonstrate that *M. suis* infection results in activation of EC.

Another of the more intriguing findings of the current study was the observation of cardiac muscle damage with disorganized and damaged and/or destroyed cardiac muscles cells. These effects could be explained by RBC aggregation in the blood vessels of *M. suis*-infected pigs with subsequent occlusion of capillaries leading to ischemia (interruption of the blood supply). This would be consistent with the fact that some cases of acute *M. suis* infection in pigs result in death within a few days [1].

In conclusion, we report several novel findings of infection with HM leading to widespread endothelial damage, RBC adhesion to the endothelium, and vascular occlusion. These vascular alterations lead to the development of hemorrhage and organ failure. To our knowledge, this is the first demonstration that HM interacts with host cells other than RBCs. In addition, the ability of *M. suis* to form biofilm-like microcolonies on the endothelium, which may protect the organism from antimicrobial agents and host immune factors, may contribute to the persistence of HM infections.

## Abbreviations

RBC: Red blood cells; Bc: Bile canaliculi; BM: Bacterial microcolony; Co: Collagen; EC: Endothelial cells; ER: Endoplasmic reticulum; F: Small fibrils; Fi: Fibrinogen fibers; IAP: Infectious anemia in pigs; ICAM 1: Intercellular adhesion molecule 1; L: Lumen; Leu: Leukocytes; LC: Lightcycler; Mi: Mitochondria; MV: Microvilli; N: Nucleus; PAEC: Porcine aortic endothelial cells; PECAM-1: Platelet endothelial cell adhesion molecule 1; Ps: Pseudopodia; PV: Plasma lemma vesicle; RM: Tunica media; SEL: Subendothelial layer; SP: Silver precipitates; V: Intracellular vacuoles; VC: Vacuolar cell; VW: Vessel wall.

## Competing interests

The authors declare that they have no competing interests.

## Authors’ contributions

Conceived and designed the experiments: LEH, UZ, KH; performed animal experiments: LEH, KH, KMF; performed microscopic experiments: AS, KG, WMA, JMM; performed in vitro analyses and data analysis: AS, MKJS, KMF, KG; wrote the manuscript: AS, KH, LEH. All authors read and approved the manuscript.

## Supplementary Material

Additional file 1 ***M. suis *infection results in perforation of the blood vessels.** Light microscopic images of the luminal surface of the aortic vessel. A. Aortic vessel of an infected pig showing RBCs attached to ECs. Scale bar = 5 mm; B. Aortic vessel of an infected control pig showing a smooth endothelial surface. Scale bar = 5 mm. Abbreviations: CT, connective tissue; I, injured endothelium with bleeding into the tissue; L, lumen; and VW, vessel wall. Click here for file

Additional file 2 ***M. suis *infection causes endothelial cell activation.** Scanning electron micrograph of an aortic vessel from an *M. suis*-infected pig. A. Aortic vessel from an infected pig showing RBCs attached to ECs. ECs (white arrows) are characterized by extensive microvilli (MV) formation. Image also shows the exposed subendothelial layer (SEL) due to detachment of ECs. Scale bar = 10 μm. Abbreviations: EC, endothelial cell; MV, microvilli; RBC, red blood cell; and SEL, subendothelial layer. Click here for file
