# Supplementary figures and images for: Mycoplasma suis infection results endothelial cell damage and activation: new insight into the cell tropism and pathogenicity of hemotrophic mycoplasma
Source: Vet Res. 2013 Feb 11;44(1):6. doi: 10.1186/1297-9716-44-6 (PMC3575358; doi:10.1186/1297-9716-44-6)

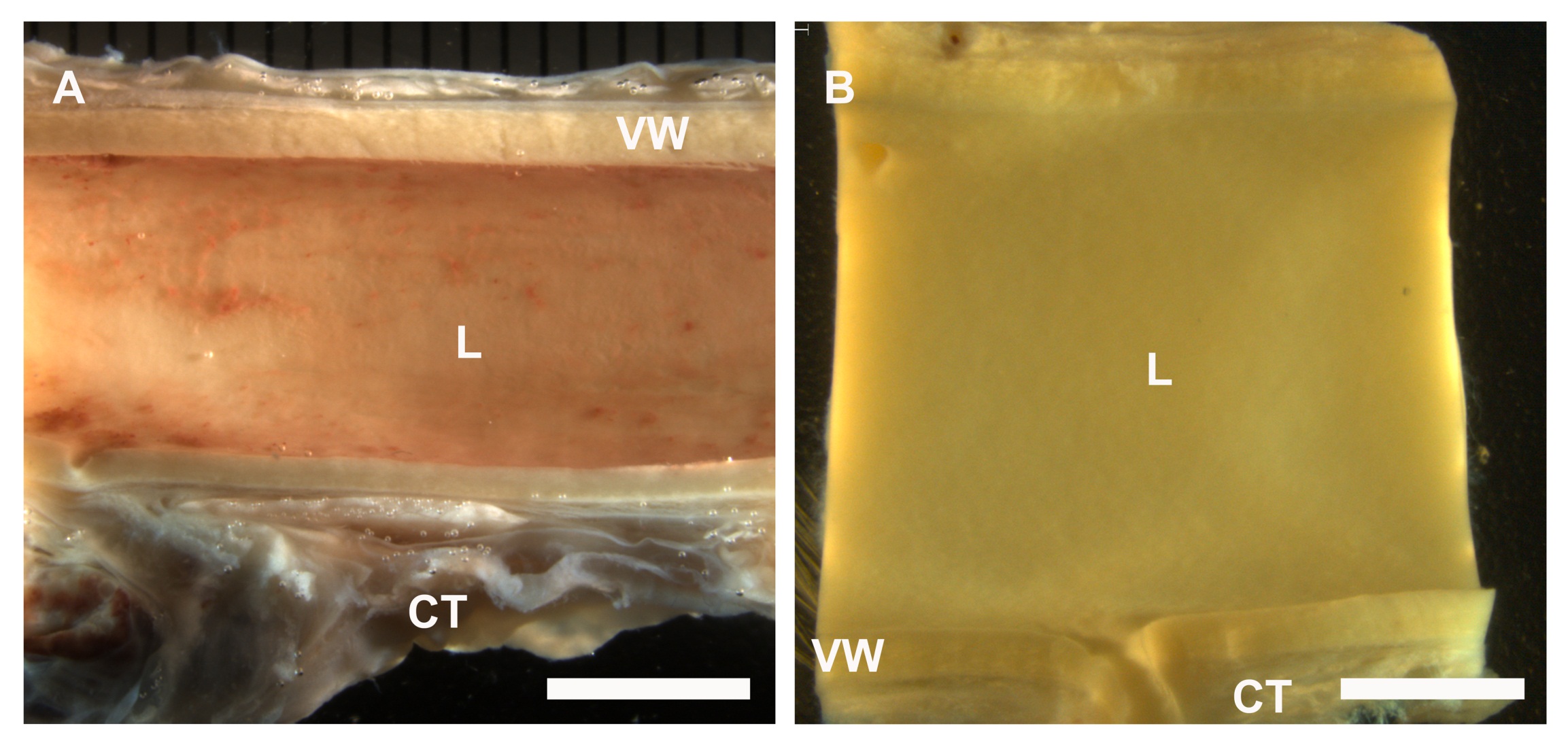

Supplement: Additional file 1 — M. suis infection results in perforation of the blood vessels. Light microscopic images of the luminal surface of the aortic vessel. A. Aortic vessel of an infected pig showing RBCs attached to ECs. Scale bar = 5 mm; B. Aortic vessel of an infected control pig showing a smooth endothelial surface. Scale bar = 5 mm. Abbreviations: CT, connective tissue; I, injured endothelium with bleeding into the tissue; L, lumen; and VW, vessel wall. [file 1297-9716-44-6-S1.doc]

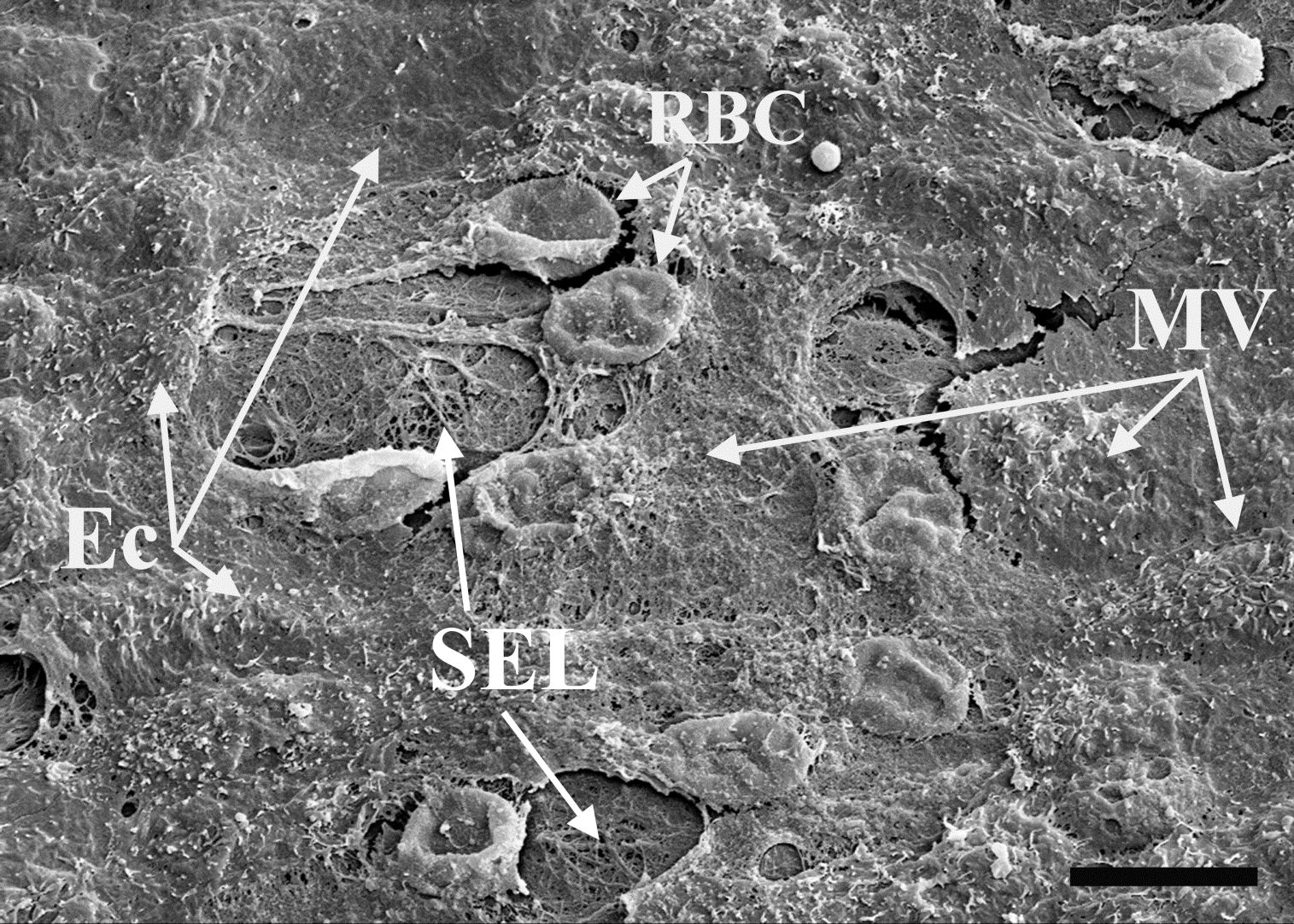

Supplement: Additional file 2 — M. suis infection causes endothelial cell activation. Scanning electron micrograph of an aortic vessel from an M. suis-infected pig. A. Aortic vessel from an infected pig showing RBCs attached to ECs. ECs (white arrows) are characterized by extensive microvilli (MV) formation. Image also shows the exposed subendothelial layer (SEL) due to detachment of ECs. Scale bar = 10 μm. Abbreviations: EC, endothelial cell; MV, microvilli; RBC, red blood cell; and SEL, subendothelial layer. [file 1297-9716-44-6-S2.doc]
